# Brain tropism acquisition: the spatial dynamics and evolution of a measles virus collective infectious unit that drove lethal subacute sclerosing panencephalitis

**DOI:** 10.1038/s44298-023-00014-0

**Published:** 2024-01-02

**Authors:** Aicha Hanna

**Affiliations:** Nature npj Viruses, https://www.nature.com/npjviruses/

## Abstract

Acute viral infections are typically cleared by the host’s immune system, but certain RNA viruses can establish ‘within host’ persistent infections, for example in the central nervous system (CNS). Neurons within the CNS are a potential site for viral persistence due to the limited capacity of the host to deploy cytolytic and inflammatory defenses in this environment. Subacute sclerosing panencephalitis (SSPE) is a rare but fatal disease caused by persistent infection with measles virus (MeV), often occurring years after acute measles. Despite the availability of effective vaccines, SSPE remains a concern due to vaccine hesitancy and disruptions in vaccination programs.

Recently, a new study by Yousaf et al.^1^ has provided insights into the spatial dynamics of MeV brain tropism acquisition by analyzing high-coverage sequencing data from multiple regions of an SSPE-affected brain.

The researchers conducted a comprehensive analysis of MeV RNA from 15 distinct brain regions of an individual who succumbed to SSPE. They utilized deep sequencing and single-cell analysis to cover the 15,894 bases of MeV genome extensively, achieving coverage of 0.89 million times. The study was retrospective, examining autopsy material donated to the Center for Disease Control and Prevention. The sequencing data were used to identify single nucleotide variants and reconstruct viral variants across different brain regions. This approach allowed for the identification of two major MeV genome subpopulations showing extensive spatially restricted diversification.

The study revealed robust viral replication across most brain regions, with evidence supporting the initiation of brain spread in the frontal cortex. Two distinct major MeV genome subpopulations were detected in all analyzed specimens, suggesting convergent evolution for modulation of fusogenicity. Mutations affecting the cytoplasmic tails of envelope proteins—F and hemagglutinin—fluctuated in frequency across regions, indicating their potential role as drivers of neurotropism acquisition.

The findings by Yousaf et al. underscore the complexity of MeV persistence and adaptation within the human brain. The identification of specific mutations that may drive neuropathogenesis provides targets for future therapeutic interventions. Moreover, this research highlights the importance of vaccination against measles not only to prevent acute disease but also to reduce the risk of long-term complications such as SSPE. The potential future impact includes better understanding of viral persistence mechanisms in the CNS, which could inform strategies for treating or preventing diseases like SSPE and other persistent viral infections.

*This text on an editorially selected paper was initially drafted using artificial intelligence, and then fact-checked and improved by an editor to meet Springer Nature publication standards.*

## References

[1] Yousaf, I., Hannon, W. W., Donohue, R. C., Pfaller, C. K., Yadav, K. & Dikdan, R. J. et al. Brain tropism acquisition: The spatial dynamics and evolution of a measles virus collective infectious unit that drove lethal subacute sclerosing panencephalitis. *PLoS Pathog.* **19**, e1011817 (2023). 38127684 10.1371/journal.ppat.1011817 PMC10735034

